# Palatal Perforation as a Rare Presentation of Severe Congenital Neutropenia Due to an ELANE Mutation

**DOI:** 10.7759/cureus.112907

**Published:** 2026-07-18

**Authors:** Ravi Shankar, Manideepa Maji, Savitri Singh, Nita Radhakrishnan

**Affiliations:** 1 Department of Pediatric Hematology Oncology, Post Graduate Institute of Child Health, Noida, IND; 2 Department of Pathology, Post Graduate Institute of Child Health, Noida, IND

**Keywords:** congenital neutropenia, elane-associated scn, elane mutation, elane variants, palatal perforation

## Abstract

Severe congenital neutropenia (SCN) is a rare inherited bone marrow failure syndrome characterized by profound neutropenia from early infancy, most commonly caused by heterozygous pathogenic variants in the *ELANE* gene. Affected children are predisposed to recurrent, severe bacterial and fungal infections. Palatal perforation has rarely been described in the setting of SCN.

We report a seven-month-old infant with recurrent severe infections, profound neutropenia, palatal perforation, and nasal septal necrosis. Although invasive fungal infection was strongly suspected, repeated microbiological and histopathological investigations were negative. Genetic testing confirmed *ELANE*-associated SCN. This case highlights a rare presentation of SCN and emphasizes the importance of considering inherited neutropenia in infants with recurrent infections and destructive midline lesions for early diagnosis and timely treatment.

This case highlights palatal perforation in *ELANE*-associated SCN. Recognition of such manifestations is important to avoid diagnostic delay and prompt early consideration of inherited neutropenia syndromes in infants with necrotic midline lesions.

## Introduction

Severe congenital neutropenia (SCN) is a rare inherited bone marrow failure syndrome characterized by profound neutropenia, typically presenting in early infancy with recurrent, severe bacterial and fungal infections [1]. The condition is most commonly associated with heterozygous pathogenic variants in the *ELANE* gene, which encodes neutrophil elastase and plays a critical role in granulopoiesis [1,2]. Arrest of myeloid maturation at the promyelocyte-myelocyte stage is the hallmark bone marrow finding, resulting in an absolute neutrophil count (ANC) persistently below 500 cells/mm³, and often below 200 cells/mm³, from birth or early infancy [2-4].

Children with SCN are predisposed to recurrent sinopulmonary infections, otitis media, gingivitis, mucositis, skin and soft-tissue infections, and invasive bacterial sepsis. Fungal infections, although less frequent than bacterial infections, may be life-threatening and are often suspected when necrotic lesions involve the nasal cavity, palate, or paranasal sinuses. In immunocompromised children, particularly those with neutropenia, palatal perforation is classically associated with invasive fungal infections such as mucormycosis, aspergillosis, or, less commonly, malignancy-related necrosis [1].

However, palatal perforation as a manifestation of SCN itself, in the absence of demonstrable fungal invasion, is exceedingly rare and sparsely documented [3]. Tissue ischemia, recurrent bacterial infection, impaired wound healing, and unchecked local inflammation may contribute to necrotic destruction of mucosal and bony structures in the setting of profound neutropenia. Recognition of such atypical presentations is important, as they may mimic invasive fungal disease and prompt aggressive antifungal therapy or surgical intervention, while the underlying etiology remains immunologic and hematologic.

We report an infant with *ELANE*-associated SCN presenting with palatal perforation and nasal septal necrosis, highlighting an unusual presentation that broadened the differential diagnosis of destructive midline lesions in infancy.

## Case presentation

A seven-month-old female infant, the second child born to non-consanguineous parents, presented with a one-week history of fever, diarrhea, and generalized skin rashes. She was a resident of Uttar Pradesh, India, with no significant antenatal or perinatal complications reported. There was no prior history of recurrent or severe infections, recurrent oral ulcers, or prior hospitalizations. Antenatal and perinatal history was unremarkable, with no history of delayed cord fall or omphalitis. There was no family history of recurrent infections, early infant deaths, or known hematologic disorders. Anthropometric assessment at admission revealed a weight of 6.5 kg (approximately 10th percentile; weight-for-age Z-score: −1.3), length of 70 cm (approximately 75th percentile; length-for-age Z-score: +0.7), and head circumference of 45 cm (approximately 90th percentile; head circumference-for-age Z-score: +1.3), based on the World Health Organization (WHO) growth standards. She had received all age-appropriate immunizations according to the National Immunization Schedule. The vaccinations included the Bacille Calmette-Guérin (BCG) vaccine at birth, three doses each of the oral polio, diphtheria-pertussis-tetanus, and hepatitis B vaccines. There was no history of adverse events following immunization, delayed healing or infection at the BCG inoculation site, injection-site infections, or vaccine-associated complications. On admission, the child was febrile and tachypneic, with evidence of respiratory distress requiring supplemental oxygen. Cutaneous examination revealed a well-demarcated, punched-out ulcerative lesion over the right lower limb, notable for minimal surrounding inflammation, erythema, or purulent discharge (Figure 1A).

**Figure 1:**
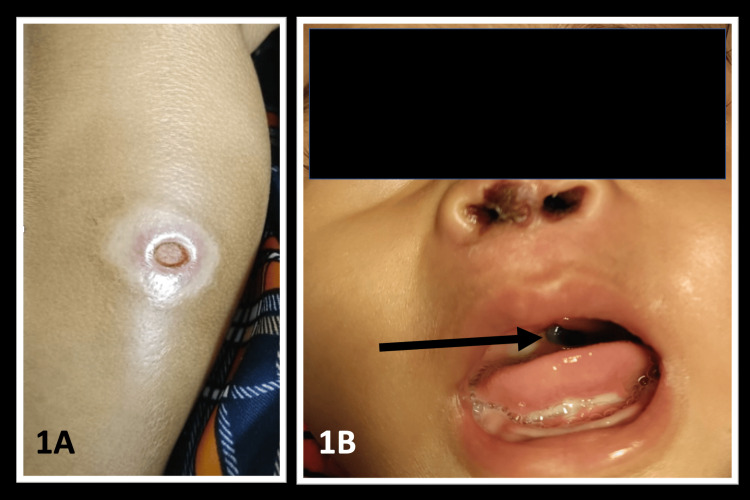
Clinical findings at presentation. (A) Punched-out necrotic ulcer over the right leg with minimal surrounding inflammation. (B) Nasal septal eschar (arrow) and hard palatal perforation (arrowhead)

The lesion appeared necrotic rather than suppurative. Additional scattered maculopapular rashes were noted over the trunk and extremities.

Initial laboratory investigations demonstrated hemoglobin of 9 g/dL, total leukocyte count of 7,210 cells/mm³, and a profoundly reduced ANC of 5 cells/mm³ and platelet count of 217,000 cells/mm^3^. Serum C-reactive protein was elevated (20 mg/L; normal <6 mg/L). Given the severity of neutropenia, a bone marrow examination was performed, which revealed marked myeloid hypoplasia with maturation arrest at the promyelocyte stage, consistent with congenital neutropenia.

Empirical intravenous meropenem and amikacin were initiated at admission. Blood cultures grew *Enterococcus faecalis*, and antimicrobial therapy was continued based on the culture sensitivity profile. The patient completed a 10-day course of intravenous meropenem and amikacin. Repeat blood cultures obtained after completion of therapy were sterile, with resolution of fever. Despite appropriate antimicrobial therapy, the child continued to exhibit persistent severe neutropenia. Recombinant granulocyte colony-stimulating factor (G-CSF) was administered; however, no sustained or meaningful rise in ANC was observed. 

Serum immunoglobulin evaluation demonstrated an elevated IgG concentration of 2469 mg/dL (age-specific reference range: approximately 200-900 mg/dL for infants aged 6-12 months). The child had not received intravenous immunoglobulin before testing. Serum IgA, IgM, and IgE concentrations were normal. The elevated serum IgG was considered reactive rather than indicative of a primary humoral immune defect. Hypergammaglobulinemia is a recognized feature in patients with SCN and other neutrophil disorders, reflecting chronic antigenic stimulation and persistent immune activation associated with recurrent or ongoing infections [5].

During the course of hospitalization, the infant developed nasal regurgitation of feeds. Careful examination of the oral cavity revealed a distinct perforation of the hard palate, with surrounding mucosal ulceration but minimal inflammatory exudate. Concurrently, she developed an eschar over the nasal septum, which subsequently sloughed off, leaving a visible defect within the nasal cavity. These findings raised a strong clinical suspicion of invasive fungal infection, particularly mucormycosis, given the profound neutropenia and necrotic midline lesions. The defect in the palate and the eschar on the nose is presented in Figure 1B. In view of persistent febrile neutropenia despite broad-spectrum antibacterial therapy and the development of necrotic lesions involving the nasal septum and hard palate, liposomal amphotericin at 3 mg/kg/day was initiated according to the febrile neutropenia protocol, which recommends mold-active antifungal therapy in high-risk patients with persistent fever and prolonged neutropenia [6].

Empirical antifungal therapy was escalated, and necrotic tissue samples were obtained for fungal staining and culture. Repeated microbiological evaluations, including fungal cultures, failed to demonstrate fungal elements. Histopathologic examination did not reveal hyphal invasion. Ear discharge culture later grew *Pseudomonas aeruginosa*, and antimicrobial therapy was modified accordingly.

Given the persistence of severe neutropenia refractory to G-CSF and the characteristic marrow findings, genetic testing for SCN was pursued. Targeted sequencing identified a heterozygous pathogenic non-sense variant in the *ELANE* gene, c.669C>A (p.Cys223*), confirming the diagnosis of *ELANE*-associated SCN.

With continued supportive care, targeted antibiotics, and optimization of nutritional status, the child’s fever subsided, and there was gradual clinical improvement. Notably, the palatal defect showed early signs of healing, with reduction in ulcer margins and improved feeding tolerance. At last follow-up, the child remained neutropenic and dependent on supportive care. Given poor response to G-CSF and the severity of infectious complications, allogeneic hematopoietic stem cell transplantation (HSCT) was advised, and donor evaluation was advised. Genetic testing was provided through the Jeffrey Modell Foundation’s “Jeffrey’s Insights” program.

## Discussion

SCN represents a spectrum of inherited disorders unified by profound neutropenia and early susceptibility to severe infections. Among these, *ELANE*-associated SCN is the most common genotype, accounting for approximately 50%-60% of cases. Pathogenic *ELANE* variants lead to misfolded neutrophil elastase, triggering endoplasmic reticulum stress and apoptosis of myeloid precursors, culminating in maturation arrest within the bone marrow [7-10].

Typical clinical manifestations of SCN include recurrent bacterial infections involving the skin, lungs, oral cavity, and middle ear, often beginning in early infancy [2]. Gingivitis, stomatitis, and aphthous ulcers are common due to impaired mucosal immunity [8]. However, destructive lesions such as palatal perforation are exceptionally uncommon and, when encountered, immediately raise concern for invasive fungal disease or malignancy [4]. Although oral mucosal inflammation is well recognized, destructive palatal lesions resulting in perforation have rarely been reported in association with SCN. Published reports of *ELANE*-associated SCN with oral involvement have predominantly described severe periodontitis, gingival inflammation, tooth mobility, and alveolar bone loss rather than palatal destruction.

Palatal perforation in immunocompromised children is most frequently associated with mucormycosis, particularly in those with prolonged neutropenia, diabetes, or corticosteroid exposure. Angioinvasion by fungal hyphae leads to tissue ischemia, necrosis, and rapid progression, often necessitating aggressive surgical debridement [7,8]. In contrast, the present case demonstrated necrotic palatal and nasal lesions without microbiological or histopathologic evidence of fungal invasion, despite extensive evaluation and high clinical suspicion. Thus, the present case expands the clinical spectrum of *ELANE*-associated SCN by documenting palatal perforation associated with profound persistent neutropenia in the absence of microbiological or histopathological evidence of invasive fungal infection. The likely mechanism is multifactorial, involving impaired neutrophil-mediated host defense, chronic bacterial colonization, tissue ischemia, and defective wound healing, rather than direct tissue invasion by an opportunistic pathogen. Recognition of this rare presentation is clinically important because destructive midline lesions are often presumed to represent invasive fungal disease, potentially delaying consideration of an underlying inherited neutropenia syndrome.

The pathophysiology of palatal perforation in SCN without fungal infection is likely multifactorial. Profound and sustained neutropenia impairs innate immune defense and compromises mucosal barrier integrity [8]. Recurrent bacterial colonization, subclinical infections, ischemic injury, and defective wound healing may collectively contribute to progressive tissue necrosis. Palatal necrosis and perforation are well-recognized complications of angioinvasive fungal infections, particularly mucormycosis and invasive aspergillosis, in children with hematological malignancies or following HSCT. Fungal invasion of blood vessel walls results in vascular thrombosis, tissue infarction, and rapidly progressive necrosis, frequently necessitating extensive surgical debridement in addition to systemic antifungal therapy [9]. In SCN, the exact pathogenesis remains uncertain but is likely multifactorial, involving profound neutropenia, impaired mucosal host defence, secondary bacterial infection, and defective tissue repair. Additionally, *ELANE*-associated neutrophil dysfunction may exacerbate local inflammatory damage even in the absence of overt infection [10,11].

Importantly, the absence of sustained response to G-CSF in this child is notable. While most patients with *ELANE*-associated SCN respond to G-CSF with improved neutrophil counts and reduced infection rates, a subset demonstrates poor or unstable responses and remains at high risk for severe infections and long-term complications, including progression to myelodysplastic syndrome or acute myeloid leukemia. In such cases, HSCT remains the only curative option [12-14].

This case underscores the importance of maintaining a broad differential diagnosis when evaluating necrotic midline lesions in neutropenic infants. While invasive fungal infection must be promptly excluded, progression of such destructive mucosal lesions, can be life threatening.

## Conclusions

Palatal perforation is an unusual and striking manifestation of severe neutropenia and may occur even without demonstration of invasive fungal infection. In infants with profound neutropenia, necrotic midline lesions should prompt thorough evaluation for infectious and malignant causes. This case highlights that destructive palatal lesions in infants with profound neutropenia should prompt concurrent evaluation for inherited neutropenia syndromes. In our patient, molecular confirmation of an *ELANE* pathogenic variant established the diagnosis of SCN, and the poor response to granulocyte colony-stimulating factor prompted planning for allogeneic HSCT, the only curative treatment for patients with severe or G-CSF-refractory disease. Early recognition and genetic diagnosis are essential to guide appropriate management, improve outcomes, and facilitate timely referral for definitive therapy.
